# Prevalence and Determinants of Adequate Compliance with Antenatal Care in Peru

**DOI:** 10.1055/s-0041-1732463

**Published:** 2021-07-27

**Authors:** Cesar Tello-Torres, Akram Hernández-Vásquez, Karla F. Dongo, Rodrigo Vargas-Fernández, Guido Bendezu-Quispe

**Affiliations:** 1 Facultad de Medicina Humana, Universidad Científica del Sur, Lima, Peru; 2 Centro de Excelencia en Investigaciones Económicas y Sociales en Salud, Vicerrectorado de Investigación, Universidad San Ignacio de Loyola, Lima, Peru; 3 Universidad Privada Norbert Wiener, Centro de Investigación Epidemiológica en Salud Global, Lima, Peru

**Keywords:** prenatal care, health surveys, cross-sectional studies, quality of health care, maternal health, maternal health services, Peru, cuidado pré-natal, inquéritos epidemiológicos, estudos transversais, qualidade da assistência à saúde, saúde materna, serviços de saúde materna, Peru

## Abstract

**Objective** To determine the adequacy of compliance with antenatal care (ANC) by pregnant women in Peru and to identify the associated factors.

**Methods** An analytical cross-sectional study of data from the 2019 Peruvian Demographic and Family Health Survey (Encuesta Demográfica y de Salud Familiar, ENDES, in Spanish) was conducted. The dependent variable was adequate compliance with ANC (provided by skilled health care professionals; first ANC visit during the first trimester of pregnancy; six or more ANC visits during pregnancy; ANC visits with appropriate content) by women aged 15 to 49 years in their last delivery within the five years prior to the survey. Crude and adjusted prevalence ratios and their 95% confidence intervals were calculated using a log-binomial regression model.

**Results** A total of 18,386 women were analyzed, 35.0% of whom adequately complied with ANC. The lowest proportion of compliance was found with the content of ANC (42.6%). Sociodemographic factors and those related to pregnancy, such as being in the age groups of 20 to 34 years and 35 to 49 years, having secondary or higher education, belonging to a wealth quintile of the population other than the poorest, being from the Amazon region, not being of native ethnicity, having a second or third pregnancy, and having a desired pregnancy, increased the probability of presenting adequate compliance with ANC.

**Conclusion** Only 3 out of 10 women in Peru showed adequate compliance with ANC. Compliance with the content of ANC must be improved, and strategies must be developed to increase the proportion of adequate compliance with ANC.

## Resumo

**Objetivo** Determinar a adequação do cumprimento dos cuidados pré-natais (CPN) por mulheres grávidas no Peru e identificar os fatores associados.

**Métodos** Foi realizado um estudo analítico transversal dos dados da Pesquisa Demográfica e de Saúde da Família Peruana de 2019 (Encuesta Demográfica y de Salud Familiar, ENDES, em espanhol). A variável dependente foi conformidade adequada com a CPN (fornecida por profissionais de saúde qualificados; primeira visita CPN durante o primeiro trimestre de gravidez; seis ou mais visitas CPN durante a gravidez; visitas CPN com conteúdo apropriado) por mulheres de 15 a 49 anos em seu último parto nos cinco anos anteriores à pesquisa. Os índices de prevalência bruta e ajustada e seus intervalos de confiança de 95% foram calculados usando um modelo de regressão log-binomial.

**Resultados** Foi analisado um total de 18.386 mulheres, das quais 35,0% cumpriram adequadamente o CPN. A menor proporção de conformidade foi encontrada com o conteúdo de ANC (42,6%). Fatores sociodemográficos e aqueles relacionados à gravidez, como estar na faixa etária de 20 a 34 anos e 35 a 49 anos, ter educação secundária ou superior, pertencer a um quintil de riqueza da população que não a mais pobre, ser da região da selva, não ser de etnia nativa, ter um segundo ou terceiro gravidez, e tendo uma gravidez desejada, aumentou a probabilidade de apresentar conformidade adequada com CPN.

**Conclusão** Apenas 3 em cada 10 mulheres no Peru mostraram conformidade adequada com o CPN. O cumprimento do conteúdo do CPN deve ser melhorado, e estratégias devem ser desenvolvidas para aumentar a proporção de cumprimento adequado com o CPN.

## Introduction

Maternal mortality still occurs in different parts of the world, despite a marked reduction from 385 to 216 deaths per 100 thousand births between 1990 and 2015. 1 Therefore, the United Nations (UN) Sustainable Development Goals (SDGs) are a blueprint established with the aim of reducing global maternal mortality to a figure lower than 70 per 100 thousand births, especially in low and middle-income countries, which account for almost all deaths. 2

Antenatal care (ANC) is considered vital to reduce maternal and neonatal morbidity and mortality. 3 4 According to the World Health Organization (WHO), ANC includes the treatment of pregnancy symptoms, nutritional consultations, evaluations of the mother and fetus, and improvement in health care services directed to the mother and the fetus. 3 Nonetheless, the literature consulted shows differences among countries regarding the number of ANC visits and compliance with ANC. 5 6 7

Latin America and the Caribbean are among the regions with the highest maternal mortality. 1 However, the number of deaths in these regions has decreased in recent decades (from 124 to 69 per 100 thousand live births in Latin America, and from 276 to 175 per 100 thousand births in the Caribbean). 1 Nevertheless, no country in this region has reached the goal of reducing maternal mortality by 75%. 1 In the case of Peru, maternal mortality has decreased from 265 to 68 per 100 thousand live births between 1990 and 2015. 1 Despite this clear reduction, in 2019, the maternal mortality rate in Peru was 56.1 per 100 thousand births, 8 indicating a scenario in which pregnancy-related deaths remain a public health problem.

The Peruvian Ministry of Health (Ministerio de Salud, MINSA, in Spanish) has established that skilled health care professionals perform adequate ANC with at least six ANC visits, the first of which is made during the first trimester of pregnancy. 9 ANC includes guidance and counseling for pregnant women, tests to support diagnosis and prophylaxis, prenatal stimulation, individual psychological consultation, dental consultation, nutrition consultation, as well as social services and legal consultations. 9 To date, adequate compliance with ANC according to the MINSA and WHO recommendations has not been studied among pregnant women in Peru. Therefore, the objective of the present study was to determine the adequacy of compliance with ANC and to identify factors associated with compliance in Peru.

## Methods

### Study Design and Population

A cross-sectional and analytical study of the data of women and their last delivery within the five years preceding the completion of the 2019 ENDES was conduct. The present manuscript was written following the guidelines of the Strengthening the Reporting of Observational Studies in Epidemiology (STROBE) statement. 10

According to its geographic characteristics, Peru is divided into three regions differentiated by geographic, climatic, and sociodemographic aspects. The Coast region borders the Pacific Ocean, and it is where Lima, the capital of the country, is located. The Andean region has the highest levels of altitude and the population with the lowest level of wealth in the country. Lastly, the Amazon is the region with the greatest biodiversity; however, its population does not have adequate access to health services due to geographic limitations regarding access. 11

The ENDES is a population-based survey conducted by the National Institute of Statistics and Informatics (Instituto Nacional de Estadística e Informática, INEI, in Spanish), which provides information on the sociodemographic and health characteristics of the population. It uses complex sampling in two stages: the first is the selection of clusters, and the second, of households. It is representative of urban and rural areas throughout Peru according to the geographic (Coast, Andean, and Amazon) and administrative regions. The ENDES uses direct interviews to collect data, and these are performed by trained personnel who visit the selected homes to fill out three questionnaires (a household questionnaire, for households and their members; an individual questionnaire, for all women of childbearing age; and a health questionnaire applied to the head of the household, which collects information on household characteristics, for persons 15 years of age or older). Detailed information on the sampling, collection, and processing of ENDES data are available on the INEI web site. 12

### Variables and Measurements

The dependent variable was adequate ANC compliance in the last pregnancy by women aged 15 to 49 years within the 5 years preceding the date of the survey. The ANC was considered adequate when fulfilling the following aspects: performed by a skilled healthcare personnel (doctors, nurses, or midwives, as reported in the ENDES data); 2 first visit before the end of the first trimester of pregnancy; six or more visits during pregnancy; and visits including all of the required services (with appropriate content). Non-completion of these aspects was considered as inadequate compliance. These components of adequate compliance with ANC have been previously used by other studies in the literature. 5 6 7 The content of the ANC visits evaluated was based on the WHO recommendations, 3 considering only the data of pregnant women participating in the survey (the WHO recommends including the measurement of blood pressure; urine and blood analysis, HIV and syphilis testing, administration of iron tablets, protection against tetanus, and information on pregnancy complications and where to go if they occur). The absence of any of these features in ANC visits was considered as non-compliance. For the present study, the minimum number of ANC visits required was six, as established by the MINSA in 2013 for the care of pregnant women in Peru. 9

According to the literature, the independent variables considered to be associated with adequate compliance with ANC 5 6 7 13 are: maternal age ([V012]: 15to 19 years; 20 to 34 years; 35 to 49 years); level of schooling ([V106]: no education or primary education; secondary education; higher education); wealth quintile ([V190]: very poor; poor; intermediate; rich; richest); geographic region (SHREGION: Metropolitan Lima; rest of the Coast; Andean; Amazon); area of residence ([V025]: urban; rural); having public health insurance ([S413]: yes; no); ethnic self-identification ([V131]: non-native; native); birth order ([BORD]: first birth; second or third births; ≥ fourth birth); desired pregnancy ([M10]: yes; no); and type of pregnancy (B0: multiple; single).

### Statistical Analysis

The 2019 ENDES databases were imported, combined, and analyzed using the Stata (StataCorp., LLC, College Station, TX, US) software, version 16. In every analysis, the weighting factors and specifications of the complex sample design of the ENDES were considered, using the *svy* command in the Stata software. Likewise, values of *p* < 0.05 were considered statistically significant for all statistical tests.

Sociodemographic characteristics and those related to the last pregnancy of the study population were reported using absolute frequencies and weighted proportions for the categorical variables, and averages with standard deviations for the numerical variables. Likewise, the spatial distribution of adequate compliance with ANC was represented according to the administrative regions of Peru (HV023: [24 departments and the constitutional province of Callao]).

To evaluate the association of the sociodemographic variables and those related to the last pregnancy with adequate compliance with ANC, prevalence ratios (PRs) and their 95% confidence intervals (95%CIs) were calculated using a log-binomial regression model. Finally, a multivariate analysis was performed to estimate the adjusted PR (aPR) for all independent variables with statistically significant values in the crude analysis.

## Results

**Fig. 1:**
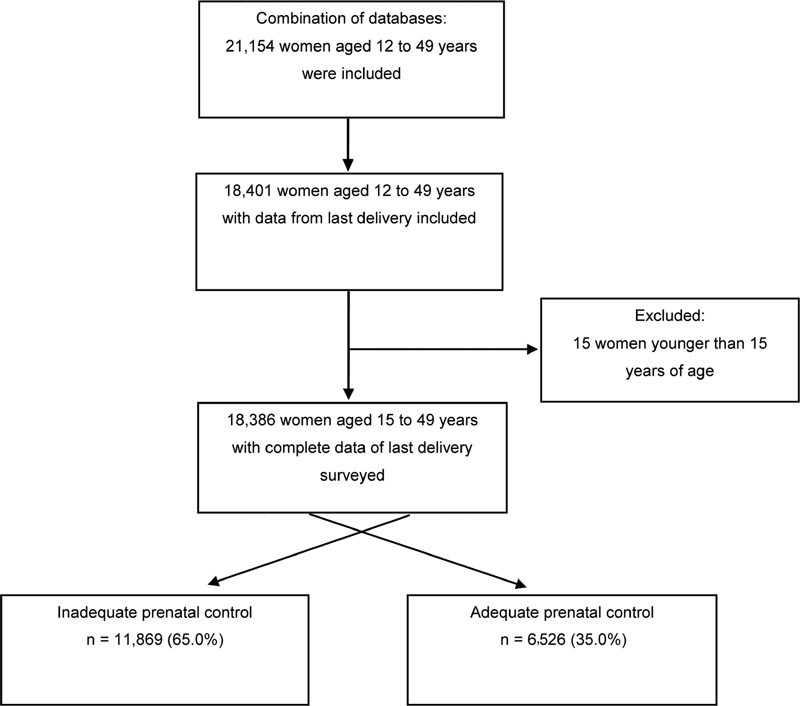
Flowchart of the selection of the study sample.

The data of 18,386 women who had delivered a child in the 5 years preceding the study were analyzed ( Fig. 1 ).

Regarding the sociodemographic characteristics of the women analyzed ( Table 1 ), 45.7% had a secondary education as their level of schooling, and 18.7% had no education or had only reached the primary level. Most of the women lived in the Coast region (56.2%). In addition, 74.8% lived in urban areas. Regarding health insurance, 67.2% had comprehensive health insurance (seguro integral de salud, SIS, in Spanish). In relation to ethnic self-identification, 6.3% reported belonging to a native ethnic group. Regarding pregnancy, 33.6% declared that they had had their first delivery; half of the women reported having had 2 or 3 deliveries, and 46.2% said that their last birth was an unwanted pregnancy. With respect to the type of pregnancy, less than 1% (0.9%) had a twin pregnancy.

**Table 1:** Characteristics of Peruvian women between the ages of 15 and 49 included in the 2019 ENDES survey

Characteristics	Absolute frequency ( *n* = 18,386)	Weighted proportion ^*^ (95%CI)	Inadequate ANC (95%CI)	Adequate ANC (95%CI)	*p* -value ^**^
*Age group (years)*					
15–19	921	4.8 (4.4–5.3)	73.7 (69.8–77.2)	26.3 (22.8–30.2)	< 0.001
20–34	12,090	65.0 (64.0–65.9)	63.7 (62.5–64.9)	36.3 (35.1–37.5)	
35–49	5,375	30.2 (29.3–31.1)	66.6 (64.8–68.4)	33.4 (31.6–35.2)	
*Level of schooling*					
No education / Primary education	3,533	18.7 (17.9–19.5)	71.7 (69.6–73.7)	28.3 (26.3–30.4)	< 0.001
Secondary education	8,576	45.7 (44.6–46.7)	62.8 (61.3–64.2)	37.2 (35.8–38.7)	
Higher education	6,277	35.6 (34.6–36.7)	64.4 (62.8–66.0)	35.6 (34.0–37.2)	
*Wealth quintile*					
Quintile 1 (lowest)	4,845	23.2 (22.3–24.1)	71.2 (69.4–73.0)	28.8 (27.0–30.6)	< 0.001
Quintile 2	5,062	24.7 (23.7–25.7)	62.7 (60.7–64.7)	37.3 (35.3–39.3)	
Quintile 3	3,735	19.9 (19.1–20.7)	60.4 (58.3–62.5)	39.6 (37.5–41.7)	
Quintile 4	2,787	17.5 (16.6–18.4)	62.7 (60.0–65.3)	37.3 (34.7–40.0)	
Quintile 5 (highest)	1,957	14.8 (14.0–15.6)	68.3 (65.3–71.1)	31.7 (28.9–34.7)	
*Geographic region*					
Coast	7,908	56.2 (55.1–57.3)	63.0 (61.4–64.5)	37.0 (35.5–38.6)	< 0.001
Andean	5,988	26.9 (25.7–28.1)	74.9 (73.2–76.4)	25.1 (23.6–26.8)	
Amazon	4,490	16.9 (16.0–17.9)	56.3 (54.1–58.4)	43.7 (41.6–45.9)	
*Residence area*					
Urban	13,230	74.8 (74.0–75.6)	63.3 (62.0–64.5)	36.7 (35.5–38.0)	< 0.001
Rural	5,156	25.2 (24.4–26.0)	70.3 (68.3–72.2)	29.7 (27.8–31.7)	
*Pulic health insurance*					
Not	5,191	32.8 (31.8–33.8)	68.1 (66.2–70.0)	31.9 (30.0–33.8)	< 0.001
Yes	13,195	67.2 (66.2–68.2)	63.5 (62.3–64.7)	36.5 (35.3–37.7)	
*Ethnicity*					
Non-native	16,808	93.7 (93.1–94.3)	64.2 (63.1–65.3)	35.8 (34.7–36.9)	<0.001
Native	1,578	6.3 (5.7–6.9)	77.0 (73.8–79.9)	23.0 (20.1–26.2)	
*Birth order*					
1	6,033	33.6 (32.7–34.4)	63.1 (61.4–64.8)	36.9 (35.2–38.6)	0.001
2 to 3	9,146	50.0 (49.1–50.9)	65.2 (63.8–66.5)	34.8 (33.5–36.2)	
≥ 4	3,207	16.4 (15.8–17.1)	68.5 (66.3–70.6)	31.5 (29.4–33.7)	
*Desired pregnancy*					
Yes	8,470	46.2 (45.2–47.1)	61.2 (59.7–62.7)	38.8 (37.3–40.3)	< 0.001
No	9,916	53.8 (52.9–54.8)	68.3 (67.0–69.6)	31.7 (30.4–33.0)	
*Type of pregnancy*					
Multiple	168	0.9 (0.7–1.0)	63.9 (54.6–72.3)	36.1 (27.7–45.4)	0.801
Single	18,218	99.1 (99.0–99.3)	65.0 (64.0–66.1)	35.0 (33.9–36.0)	

Abbreviation: CI, confidence interval; ENDES, Encuesta Demográfica y de Salud Familiar (Demographic and Family Health Survey).

Notes: ^*^ The weighting factor and sample specifications of the 2019 ENDES were included; ^**^ the p-value was calculated using the Chi-squared test.

**Fig. 2:**
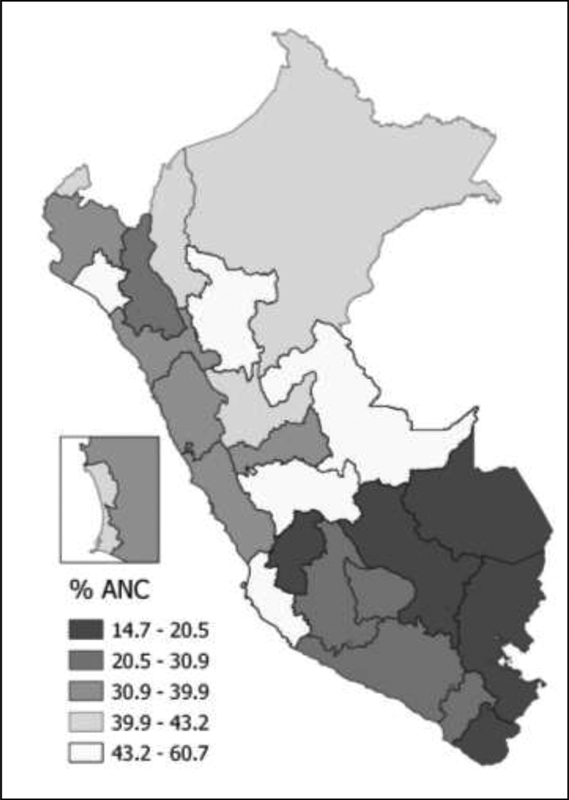
Proportion of compliance with the antenatal care (ANC) components among Peruvian women by region, according to the 2019 Peruvian Demographic and Family Health Survey (Encuesta Demográfica y de Salud Familiar, ENDES, in Spanish).

The ANC visits were adequate in 35.0% of the women analyzed ( Table 2 ). Based on the administrative regions of the Peruvian territory, in general, the lowest proportions of ANC visits were found in the Andean region ( Fig. 2 ). According to the services included in adequate ANC, almost all women (98.3%) were cared for by skilled health care personnel. The first ANC visit was in the first trimester of pregnancy for 81.10%, and 89.70% underwent six or more visits. Regarding compliance with the content of ANC as stipulated by the MINSA, 42.6% of the women underwent ANC visits with appropriate content.

**Table 2:** Proportion of compliance with the antenatal care (ANC) components among Peruvian women according to the 2019 ENDES

Characteristic	Absolute frequency ( *n* = 18,386)	Weighted proportion* (95% confidence interval)
Care for by skilled health care personnel	18,042	98.3 (97.9–98.6)
Six antenatal care visits or more	16,499	89.7 (89.1–90.3)
First antenatal care visit within first trimester	14,768	81.1 (80.3–81.9)
Antenatal care with appropriate content	7,921	42.6 (41.5–43.7)
All four characteristics	6,526	35.0 (33.9–36.0)

Abbreviation: ENDES, Encuesta Demográfica y de Salud Familiar (Demographic and Family Health Survey).

Note: ^*^ The weighting factor and sample specifications of the 2019 ENDES were included.

In relation to the analysis of the association between sociodemographic variables and adequate compliance with ANC ( Table 3 ), we found that women in the age groups of 20 to 34 years (aPR: 1.38; 95%CI: 1.19–1.60) and from 35 to 49 years (aPR: 1.36; 95%CI: 1.16–1.61) had a higher probability of presenting adequate compliance with ANC compared with adolescent pregnant women. Regarding the level of schooling, women with secondary (aPR: 1.19; 95%CI: 1.10–1.29) or higher education (aPR: 1.17; 95%CI: 1.06–1.30) had a higher probability of having adequate ANC compared with those with no education or only primary education. According to the wealth quintile, poorer women (quintile 1) were generally less likely to have had appropriate ANC compared with those in quintile 2 (aPR: 1.12; 95%CI: 1.02–1.23) and quintile 3 (aPR: 1.18; 95%CI: 1.06–1.31). As for the geographic domain, in the Andean region, women were less likely to receive proper ANC compared with those from the Coast region (aPR: 0.73; 95%CI: 0.67–0.79), while women from the Amazon region were more likely to have adequate ANC compared with those from the Coast (aPR: 1.26; 95%CI: 1.17–1.35). No significant differences were found between women in rural and urban areas concerning the probability of presenting adequate compliance with ANC ( *p* = 0.188). Regarding ethnic self-identification, women of native ethnicity were less likely to have adequate ANC (aPR: 0.83; 95%CI: 0.71–0.96).

**Table 3:** Factors associated with adequate compliance with antenatal care among women who received prenatal care for the last birth according to the 2019 ENDES

Characteristic	Bivariate model		Adjusted model*	
	PR (95%CI)	*p* -value	aPR (95%CI)	*p* -value
*Age group (years)*				
15–19	Ref.		Ref.	
20–34	1.38 (1.20–1.59)	< 0.001	1.38 (1.19–1.60)	< 0.001
35–49	1.27 (1.09–1.47)	0.002	1.36 (1.16–1.61)	< 0.001
*Level of schooling*				
No education / Primary education	Ref.		Ref.	
Secondary education	1.31 (1.21–1.42)	< 0.001	1.19 (1.10–1.29)	< 0.001
Higher education	1.26 (1.15–1.37)	< 0.001	1.17 (1.06–1.30)	0.002
*Wealth quintile*				
Quintile 1 (lowest)	Ref.		Ref.	
Quintile 2	1.30 (1.19–1.41)	< 0.001	1.12 (1.02–1.23)	0.021
Quintile 3	1.38 (1.27–1.49)	< 0.001	1.18 (1.06–1.31)	0.002
Quintile 4	1.30 (1.18–1.43)	< 0.001	1.13 (1.00–1.28)	0.058
Quintile 5 (highest)	1.10 (0.99–1.23)	0.085	1.00 (0.87–1.16)	0.948
*Geographic region*				
Coast	Ref.		Ref.	
Andean	0.68 (0.63–0.73)	< 0.001	0.73 (0.67–0.79)	< 0.001
Amazon	1.18 (1.11–1.26)	< 0.001	1.26 (1.17–1.35)	< 0.001
*Residence area*				
Urban	Ref.		Ref.	
Rural	0.81 (0.75–0.87)	< 0.001	0.94 (0.85–1.03)	0.188
*Public health insurance*				
No	Ref.		Ref.	
Yes	1.14 (1.07–1.22)	< 0.001	1.25 (1.16–1.34)	< 0.001
*Ethnicity*				
Non-native	Ref.		Ref.	
Native	0.64 (0.56–0.74)	< 0.001	0.83 (0.71–0.96)	0.010
*Birth order*				
1	Ref.		Ref.	
2 to 3	0.94 (0.89–1.00)	0.052	0.92 (0.87–0.98)	0.006
≥ 4	0.85 (0.79–0.93)	<0.001	0.92 (0.84–1.01)	0.070
*Desired pregnancy*				
Yes	Ref.		Ref.	
No	0.82 (0.78–0.86)	<0.001	0.82 (0.78–0.86)	< 0.001
*Type of pregnancy*				
Multiple	Ref.		Not included	
Single	0.97 (0.76–1.24)	0.800		

Abbreviations: aPR, adjusted prevalence ratio; CI, confidence interval; PR, prevalence ratio.

Note: ^*^ A generalized linear model of the binomial family was performed for complex samples. Variables with a *p* value < 0.05 in the bivariate analysis were included.

Regarding the variables related to the last pregnancy and their association with adequate compliance with ANC ( Table 3 ), women in older age groups had a higher probability of presenting adequate compliance with the ANC visits. A significant difference was observed between women with a second or third birth compared with those delivering for first time (aPR: 0.92; 95%CI: 0.87–0.98). There was no difference in adequate compliance with ANC between women who had a first birth or those with four or more births ( *p* = 0.070). Women with an unwanted pregnancy had a lower probability of adequate compliance with ANC (aPR: 0.82; 95%CI: 0.78–0.86). On the other hand, there were no differences between women who had a single birth compared with those with multiple births in relation to adequate compliance with ANC ( *p* = 0.800).

## Discussion

Antenatal care is a strategy that is promoted throughout the world to improve maternal health, and it is included in the Peruvian regulations for the adequate care of pregnant women. However, the results of the present study indicate that less than half of pregnant women in Peru received adequate ANC during the five years prior to the 2019 ENDES survey. The component with the lowest ANC compliance was the provision of the complete ANC content (care by skilled health care personnel, the first visit made before the end of the first trimester of pregnancy, and six or more ANC visits during pregnancy). Likewise, the sociodemographic factors, such as age, level of schooling, well-being index, ethnicity, and region of origin, as well as factors related to pregnancy, such as the order of the number of deliveries and desired pregnancy, were associated with non-compliance with ANC.

The 2019 ENDES showed that only 3 out of 10 pregnant Peruvian women presented adequate compliance with ANC, and the lowest proportion of compliance was regarding the fulfillment of all the content required to receive an adequate ANC (4 out of 10). In a study performed in 2019, 14 1 in 2 pregnant women received ANC with adequate compliance to the content, which is similar to the findings of the present study. Similarly, in low and middle-income countries, low proportions of compliance with the contents of ANC have been reported. 5 6 7 In relation to maternal health, in recent decades, there has been an increase in the number of ANC visits per pregnant women, as well as in ANC provided by skilled health care personnel. 15 Likewise, in Peru, ANC provided by skilled health care personnel has increased from 54.6% (1986) to 98.2% (2019). 14 These increases are consistent with the global panorama, which reveals an increase in the number of ANC visits, as well as in the proportion of women who start ANC early, and in the care provided by skilled health care personnel. 15 Given the widely-studied relationship between inadequate ANC and the presence of negative maternal and perinatal outcomes, such as maternal and fetal death or low birth weight, 16 17 it is necessary to strengthen ANC in Peru. Improvements in ANC should include not only sustaining the rise in the number of pregnant women cared for by skilled health care personnel with an adequate initiation and number of ANC visits, but also strategies to enable the recruitment of pregnant women who are not yet receiving this care, as well as to improve compliance with ANC content during pregnancy.

In relation to sociodemographic factors, we found that the higher the level of education, the more likely the mother was to present adequate compliance with ANC, and that women with a higher level of schooling were more likely to adequately comply with ANC visits, which is similar to what has been described worldwide. 5 6 7 18 19 20 It has been observed that women with a higher level of schooling tend to value more the ANC, which would explain the greater adequate compliance with ANC in this group. Similarly, several studies 5 6 7 21 have shown that women with a higher socioeconomic status are more likely to receive adequate ANC and even a larger number of services. In Peru, the SIS provides free public insurance that covers ANC visits and childbirth for every woman in the Peruvian territory who does not have another type of health insurance. One of the findings of the present study was that women with SIS showed a higher proportion of adequate compliance with ANC. The results of the present study describe a scenario in which, despite ANC visits and ANC content being covered and regulated for all women in the Peruvian territory, adequate ANC differs according to the level of schooling and the socioeconomic level, as well as having health insurance.

Regarding the geographic domain, women from the Andean region were less likely to adequately comply with ANC compared with those from the Coast. In the five years preceding the study, the proportion of women seen in the first trimester increased; however, in 2018, a lower proportion of women from the Andean region had their first ANC visit during this period compared with those from the Coast region (74.8% versus 84.6%, respectively). 22 On the other hand, women from the Amazon region were more likely to have ANC with adequate compliance compared with those from the Coast region. Similarly to the Andean region, there has been an increase in the proportion of women in the Amazon region undergoing ANC visits in recent years, as well as in the number of those who started ANC during the first trimester of pregnancy. Nonetheless, Peruvian women from the Andean and Amazon regions have the highest proportion of maternal mortality in the country; 8 23 therefore, increased efforts are needed to develop strategies to reduce this health indicator.

There were no differences between women living in rural areas and those living in urban areas in relation to less adequate compliance with ANC. In recent years, the proportion of rural women who have undergone an ANC visit during the first trimester has increased, in addition to the number of visits received and other useful strategies to reduce maternal mortality, such as institutionalized delivery. 12 However, there is still a gap between women in urban and rural areas regarding ANC. 12 A similar situation has been observed throughout the world, 15 and it has been reported that rurality is associated with an increase in maternal mortality. 24 Taking into account that the area of residence is related to ANC onset and delay, 25 joint efforts are needed to sustain the increase in the number of ANC visits received by women in rural areas and to achieve adequate compliance with ANC, to contribute to reduce morbidity and mortality in both the mother and the fetus.

Regarding the age of the pregnant women, the probability of having adequate ANC increased with age. Previous studies 6 7 performed in other countries report that the older the pregnant woman, the more likely she is to receive adequate ANC. A previous study 11 have reported that there was no difference between the age groups of women of childbearing age in terms of receiving the required ANC content. A possible explanation for why older women have greater adequate compliance with ANC could be that they may be considered as being at a greater risk by health care personnel, or that the experience of previous pregnancies sensitizes these women about the importance of undergoing ANC visits, thereby increasing the likelihood of adequate compliance. Regarding ethnic identification, self-identified native women were less likely to adequately comply with the ANC visits. In Latin America and the Caribbean, a lower proportion of women of native ethnic groups have access to ANC and delivery care provided by skilled health care personnel compared to women of non-native ethnic groups, 26 with reports of difficulties in the implementation of programs to increase the number of women of native ethnic groups receiving ANC. 27

In relation to the characteristics of the pregnancy, women with four or more previous deliveries had a lower probability of presenting adequate compliance with ANC. Some studies 13 28 29 performed in other countries describe that the greater the number of births a woman has had, the less likely she will attend ANC visits, possibly due to the feeling of security that the experiences of the previous births bring her. On the other hand, studies have observed that women with an unwanted pregnancy are less likely to adequately comply with ANC or to start ANC controls early, because of they have less interest in this care. These results could explain the differences found in the present study. In Peru, there is an unmet need for family planning, and women have difficulty preventing unwanted pregnancies, in addition to the social stigma of continuing with a pregnancy when they are in a socially-undesirable personal situation or undergoing an abortion 30 (which occurs at a high rate, despite being illegal in the country, except for medical reasons). Unwanted pregnancies are described in the literature 31 32 33 as associated with an increased risk of maternal and neonatal health complications, among other adverse outcomes, including a higher risk of cesarean sections, inappropriate weight gain, and mental health disorders. These characteristics associated with non-compliance with ANC indicate which population subgroups of women of childbearing age would not receive adequate ANC in accordance with the WHO recommendations and Peruvian standards, and a major concern of decision-makers is the achievement of adequate compliance with ANC in these women.

In regard to the limitations of the present study, since it is a study of secondary data, it is likely that some of the data may not be adequate. Another limitation of the study related to the ENDES is the possibility of introducing a recall bias and the lack of understanding of some questions by the respondents, a situation that is typical of any study based on surveys that collect data on past events. In addition, the ENDES does not record data on diseases or risk factors for women that could be of great use to health services, including ANC visits. However, the ENDES has protocols that ensure the quality of the recorded data, and it is widely used by public institutions and researchers as a source of information for the development of research and decision-making regarding health care in Peru. Moreover, it is a survey of national and regional representation, which records data that are used to evaluate adequate compliance with ANC based on the recommendations of the WHO and regulated by the MINSA.

## Conclusion

In conclusion, in 2019, we observed that 3 out of 10 women in Peru presented adequate compliance with ANC, as recommended by the WHO and the MINSA standards. In relation to the components of adequate compliance with ANC, only 4 out of 10 women had undergone ANC that included the required care content. In addition, population subgroups, such as women from the Andean region and rural areas, native ethnic groups, and those with lower level of schooling and socioeconomic status were less likely to present adequate compliance with ANC. These findings reveal the need to strengthen ANC among the Peruvian population, with an emphasis on providing the required content during care and developing strategies for population subgroups with a lower likelihood of having adequate compliance with ANC.

## References

[1] Trends in maternal mortality: 1990 to 2015: estimates by WHO, UNICEF, UNFPA, World Bank Group and the United Nations Population Division [Internet] Geneva World Health Organization 2015[cited 2020 Sep 30]. Available from:http://www.who.int/reproductivehealth/publications/monitoring/maternal-mortality-2015/en/

[2] World Health Organization Definition of skilled health personnel providing care during childbirth: the 2018 joint statement by WHO, UNFPA, UNICEF, ICM, ICN, FIGO and IPA [Internet] 2018[cited 2020 Sep 30]. Available from:https://apps.who.int/iris/bitstream/handle/10665/272818/WHO-RHR-18.14-eng.pdf?ua=1

[3] World Health Organization WHO recommendations on antenatal care for a positive pregnancy experience [Internet] Geneva World Health Organization 2016[cited 2020 Sep 28]. Available from:http://www.who.int/reproductivehealth/publications/maternal_perinatal_health/anc-positive-pregnancy-experience/en/28079998

[4] Kerber K J de Graft-Johnson J E Bhutta Z A Okong P Starrs A Lawn J E Continuum of care for maternal, newborn, and child health: from slogan to service delivery Lancet 2007 370(9595):1358 1369 10.1016/S0140-6736(07)61578-5 17933651

[5] Heredia-Pi I Servan-Mori E Darney B G Reyes-Morales H Lozano R Measuring the adequacy of antenatal health care: a national cross-sectional study in Mexico Bull World Health Organ 2016 94 06 452 461 10.2471/BLT.15.168302 27274597 PMC4890208

[6] Siddique A B Perkins J Mazumder T Antenatal care in rural Bangladesh: Gaps in adequate coverage and content PLoS One 2018 13 11 e0205149 10.1371/journal.pone.0205149 30452444 PMC6242304

[7] Singh L Dubey R Singh S Goel R Nair S Singh P K Measuring quality of antenatal care: a secondary analysis of national survey data from India BJOG 2019 126 04 7 13 10.1111/1471-0528.15825 31127680

[8] Gil C F Situación epidemiológica de la mortalidad materna en el Perú, SE 52. Bol Epidemiol Perú [Internet] 2019[cited 2020 Sep 29];28(52):1334–40. Available from:http://www.dge.gob.pe/portal/docs/vigilancia/boletines/2019/52.pdf

[9] Ministerio de Salud NTS No. 105: Norma Técnica de Salud para la Atención Integral de Salud Materna [Internet] 2013[cited 2020 Sep 30]. Available from:http://www.diresacusco.gob.pe/salud_individual/dais/materno/NORMAS%20RTN/03/RM827-2013%20-%20NTS%20DE%20SALUD%20MATERNA.pdf

[10] STROBE Initiative von Elm E Altman D G Egger M Pocock S J Gøtzsche P C Vandenbroucke J P Strengthening the Reporting of Observational Studies in Epidemiology (STROBE) statement: guidelines for reporting observational studies BMJ 2007 335(7624):806 808 10.1136/bmj.39335.541782.AD 17947786 PMC2034723

[11] You H Yu T Gu H Factors Associated With Prescribed Antenatal Care Utilization: A Cross-Sectional Study in Eastern Rural China. Inquiry 2019 56 4.6958019865435E13 10.1177/0046958019865435 PMC6681245 31370723

[12] Instituto Nacional de Estadística e Informática. Perú: Encuesta Demográfica y de Salud Familiar - ENDES 2019 [Internet] 2020[cited 2020 Sep 20]. Available from:https://www.inei.gob.pe/media/MenuRecursivo/publicaciones_digitales/Est/Endes2019/

[13] Abame D E Abera M Tesfay A Relationship between unintended pregnancy and antenatal care use during pregnancy in Hadiya Zone, Southern Ethiopia J Reprod Infertil 2019 20 01 42 51 30859081 PMC6386792

[14] Hernández-Vásquez A Vargas-Fernández R Bendezu-Quispe G Factores asociados a la calidad de la atención prenatal en Perú Rev Peru Med Exp Salud Publica 2019 36 02 178 187 10.17843/rpmesp.2019.362.4482 31460628

[15] UNICEF. Antenal Care [Internet] 2019[cited 2020 Sep 10]. Available from:https://data.unicef.org/topic/maternal-health/antenatal-care/

[16] Chen X K Wen S W Yang Q Walker M C Adequacy of prenatal care and neonatal mortality in infants born to mothers with and without antenatal high-risk conditions Aust N Z J Obstet Gynaecol 2007 47 02 122 127 10.1111/j.1479-828X.2007.00697.x 17355301

[17] da Fonseca C R Strufaldi M W de Carvalho L R Puccini R F Adequacy of antenatal care and its relationship with low birth weight in Botucatu, São Paulo, Brazil: a case-control study BMC Pregnancy Childbirth 2014 14 255 10.1186/1471-2393-14-255 25085236 PMC4131026

[18] Muyunda B Makasa M Jacobs C Musonda P Michelo C Higher educational attainment associated with optimal antenatal care visits among childbearing women in Zambia Front Public Health 2016 4 127 10.3389/fpubh.2016.00127 27379228 PMC4909780

[19] Babalola S Women's education level, antenatal visits and the quality of skilled antenatal care: a study of three African countries J Health Care Poor Underserved 2014 25 01 161 179 10.1353/hpu.2014.0049 24509018

[20] Afaya A Azongo T B Dzomeku V M Women's knowledge and its associated factors regarding optimum utilisation of antenatal care in rural Ghana: A cross-sectional study PLoS One 2020 15 07 e0234575 10.1371/journal.pone.0234575 32645002 PMC7347137

[21] Pandey S Karki S Socio-economic and demographic determinants of antenatal care services utilization in Central Nepal Int J MCH AIDS 2014 2 02 212 219 27621975 PMC4948147

[22] Instituto Nacional de Estadística e Informática Informe Perú: indicadores de resultados de los programas presupuestales, primer semestre 2019 elaborado con los resultados de la Encuesta Demográfica y de Salud Familiar (ENDES) [Internet] 2019[cited 2020 Sep 25]. Available from:https://proyectos.inei.gob.pe/endes/2019/ppr/Indicadores_de_Resultados_de_los_Programas_Presupuestales_ENDES_Primer_Semestre_2019.pdf

[23] dl Carpio Ancaya L [Situation of maternal mortality in Peru, 2000 - 2012] Rev Peru Med Exp Salud Publica 2013 30 03 461 464 24100823

[24] World health Organization. Maternal health [Internet] 2020[cited 2020 Oct 3]. Available from:https://www.who.int/health-topics/maternal-health#tab=tab_1

[25] Ewunetie A A Munea A M Meselu B T Simeneh M M Meteku B T DELAY on first antenatal care visit and its associated factors among pregnant women in public health facilities of Debre Markos town, North West Ethiopia BMC Pregnancy Childbirth 2018 18 01 173 10.1186/s12884-018-1748-7 29769122 PMC5956942

[26] Mesenburg M A Restrepo-Mendez M C Amigo H Ethnic group inequalities in coverage with reproductive, maternal and child health interventions: cross-sectional analyses of national surveys in 16 Latin American and Caribbean countries Lancet Glob Health 2018 6 08 e902 e913 10.1016/S2214-109X(18)30300-0 30012271 PMC6057134

[27] Comisión Económica para América Latina y el Caribe Salud materno-infantil de pueblos indígenas y afrodescendientes de América Latina: aportes para una relectura desde el derecho a la integridad cultural [Internet] 2010[cited 2020 Sep 30]. Available from:https://www.cepal.org/es/publicaciones/3797-salud-materno-infantil-pueblos-indigenas-afrodescendientes-america-latina-aportes

[28] Erol N Durusoy R Ergin I Döner B Ciçeklioğlu M Unintended pregnancy and prenatal care: a study from a maternity hospital in Turkey Eur J Contracept Reprod Health Care 2010 15 04 290 300 10.3109/13625187.2010.500424 20809676

[29] Kassahun E A Zeleke L B Dessie A A Factors associated with unintended pregnancy among women attending antenatal care in Maichew Town, Northern Ethiopia, 2017 BMC Res Notes 2019 12 01 381 10.1186/s13104-019-4419-5 31277714 PMC6612166

[30] Palomino N Padilla M R Talledo B D Mazuelos C G Carda J Bayer A M The social constructions of unwanted pregnancy and abortion in Lima, Peru Glob Public Health 2011 6 01 S73 S89 21732707 10.1080/17441692.2011.590813

[31] Bahk J Yun S C Kim Y M Khang Y H Impact of unintended pregnancy on maternal mental health: a causal analysis using follow up data of the Panel Study on Korean Children (PSKC) BMC Pregnancy Childbirth 2015 15 85 25881099 10.1186/s12884-015-0505-4 PMC4387588

[32] Dehingia N Dixit A Atmavilas Y Unintended pregnancy and maternal health complications: cross-sectional analysis of data from rural Uttar Pradesh, India BMC Pregnancy Childbirth 2020 20 01 188 32228511 10.1186/s12884-020-2848-8 PMC7106893

[33] Omani-Samani R Ranjbaran M Mohammadi M Impact of unintended pregnancy on maternal and neonatal outcomes J Obstet Gynaecol India 2019 69 02 136 141 10.1007/s13224-018-1125-5 30956467 PMC6430264

